# Accelerated Vascular Aging in Women with Prior Preeclampsia: A Review of Epidemiology, Pathophysiological Mechanisms, and Geroprotective Strategies

**DOI:** 10.3390/jcm15051880

**Published:** 2026-03-01

**Authors:** M. Yeo, D. W. Kwak, S. Y. Kim, A. Y. Choi, M. Kwak, J. I. Yang

**Affiliations:** 1 Center of Materno-Fetal Intensive Care Unit, Division of Materno-Fetal Medicine, Department of Obstetrics and Gynecology, Ajou University School of Medicine, 164 World Cup-ro, Yeongtong-gu, Suwon 16499, Gyeonggi-do, Republic of Korea; 2 Medical Research Center, H&M Co., Ltd., Yongin 18469, Gyeonggi-do, Republic of Korea

**Keywords:** preeclampsia, geroscience, vascular aging, ovarian senescence, biological age, risk stratification

## Abstract

Preeclampsia (PE) has traditionally been regarded as a pregnancy-limited hypertensive disorder; however, accumulating evidence increasingly positions it as a pivotal early-life vascular stress test that manifests underlying vulnerabilities and accelerates biological aging. Women with a history of PE exhibit a heightened susceptibility to premature-onset multi-systemic diseases, specifically cardiovascular, ovarian, renal, and metabolic decline. This suggests that PE acts as a catalyst for accelerated aging, driven by shared pathophysiological pathways that represent common mechanisms of systemic senescence. This review provides a comprehensive analysis of the epidemiological links and pathogenic drivers underpinning accelerated systemic aging following PE, with a specific focus on the cardiovascular-ovarian axis. Epidemiological data consistently demonstrate that women with prior PE exhibit significantly reduced anti-Müllerian hormone (AMH) levels, translating to an estimated 1.5-year acceleration in reproductive aging. In parallel, PE is associated with a twofold increase in lifetime cardiovascular disease (CVD) risk and the onset of chronic hypertension occurring an average of 7.7 years earlier. However, reconciling the phenotypic heterogeneity of PE and transcending the constraints of non-experimental designs are essential for firmly establishing this accelerated aging paradigm. At the molecular level, PE and ovarian aging converge on shared pathways—including mitochondrial dysfunction, oxidative stress, inflammation, and epigenetic dysregulation—collectively defining a distinct pathogenic ovarian–vascular aging axis. Proposed geroscience-based strategies advocate for refined risk stratification by incorporating molecular aging biomarkers—such as epigenetic clocks and inflammatory profiles—alongside conventional clinical indicators. This integrative framework facilitates the early identification of high-risk aging phenotypes, enabling targeted monitoring and timely interventions to preemptively modulate accelerated aging pathways. Pharmacological approaches within this framework emphasize the judicious repurposing of established agents, such as metformin, statins, and SGLT2 inhibitors, while emerging gerotherapeutics, including senolytics and senomorphics, provide a conceptual foundation for targeting the fundamental biological drivers of senescence. Although these geroprotective strategies, including the repurposing of established agents and the use of senolytics, offer innovative conceptual frameworks for targeting the fundamental drivers of senescence, they remain largely exploratory and require further clinical validation. Such strategies offer novel opportunities to shift the clinical focus from treating isolated comorbidities to modulating the shared molecular substrates of aging, ultimately promoting healthy aging and functional longevity in the elderly female population.

## 1. Introduction

**Figure 1:**
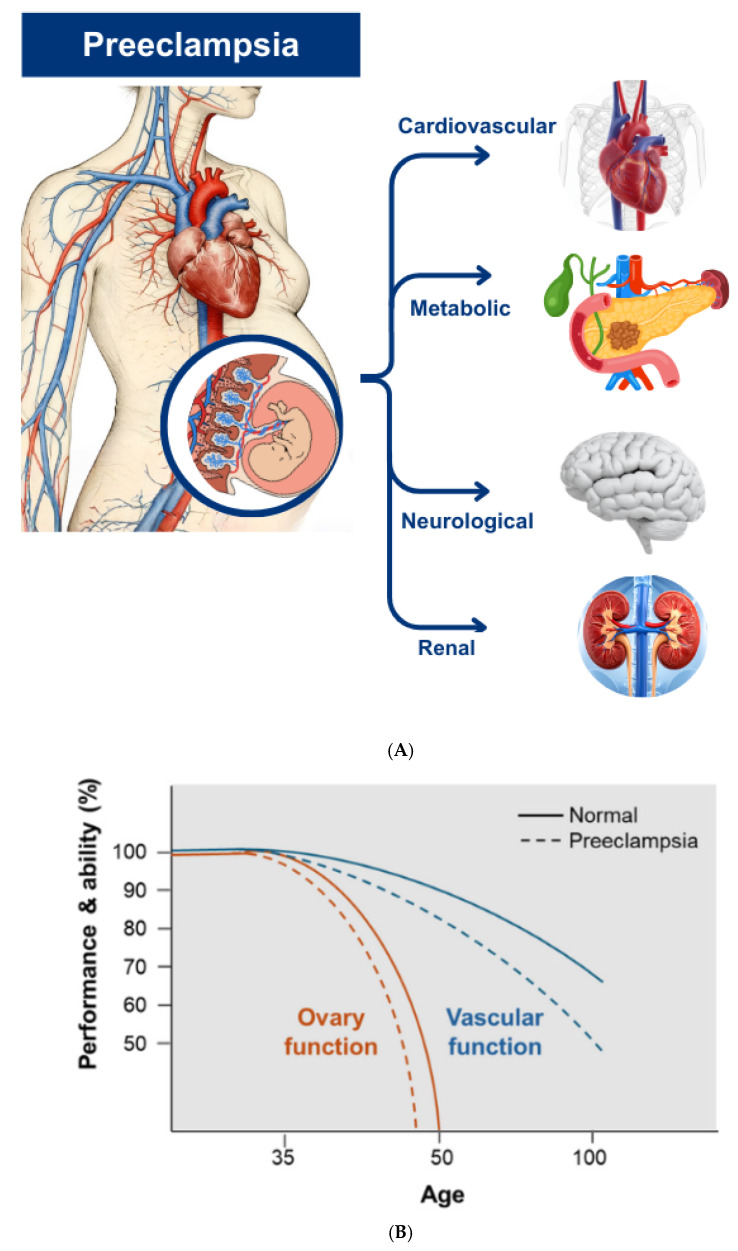
Systemic Impact of Preeclampsia on Multi-organ Aging and Functional Decline. (**A**) Systemic impact of preeclampsia. The schematic delineates the multi-organ domains affected by PE. Systemic vascular dysfunction triggers a cascade of renal, metabolic, and cardiovascular vulnerabilities, while further contributing to neurological decline and ovarian senescence, thereby positioning PE as a key driver of accelerated female biological aging. (**B**) Schematic Representation of Accelerated Functional Decline. This schematic graph compares the theoretical longitudinal trajectories of ovary and vascular functions between normal individuals (solid lines) and those with a history of PE (dashed lines). Following the insult of PE, there is a distinct downward shift in performance and ability (%), representing the acceleration of biological aging. This model illustrates a potential framework wherein PE-induced stress might shift the physiological healthspan, potentially advancing the onset of age-related functional decline within the ovarian–vascular axis.

Preeclampsia (PE), a multisystem hypertensive disorder occurring in 2–8% of pregnancies, has traditionally been managed as a transient condition that resolves upon delivery of the placenta [1,2]. However, contemporary longitudinal data have reframed PE as a critical “early-life vascular stress test” that reveals a woman’s underlying predisposition to chronic diseases [3,4,5] (Figure 1A). Emerging evidence suggests that the impact of PE extends substantially beyond the peripartum period, acting as a potent catalyst for accelerated biological aging [6,7,8] (Figure 1B). In particular, the increasing clinical emphasis on managing cardiovascular diseases within aging populations highlights the necessity of understanding how mid-life reproductive milestones, such as a history of PE, dictate the trajectory of cardiovascular health in later years.

Moving beyond established clinical associations between PE and future cardiovascular events, this review expands the current paradigm by integrating obstetric milestones into the comprehensive framework of geroscience. By synthesizing emerging molecular hallmarks of aging with longitudinal clinical data, we underscore the necessity of proactive, life-course health management. Specifically, this work introduces a unified mechanistic trajectory—the ovarian-vascular axis of senescence—to reframe PE as a critical window into a woman’s biological aging rather than a transient gestational event. This integrative perspective facilitates a novel exploration of gerotherapeutic interventions and systemic risk stratification, offering a strategic path to extend cardiovascular healthspan throughout the life course.

### 1.1. Physiological Ovarian Aging and Its Systemic Impact

Ovarian aging is well-established as the primary manifestation of organ-specific senescence in the female body, characterized by a quantitative and qualitative decline in the oocyte pool. While traditionally viewed through a reproductive lens, contemporary evidence underscores this process as a systemic event. The deterioration of ovarian function—clinically indexed by declining anti-Müllerian hormone (AMH) levels—precipitates the attrition of estrogen’s systemic cardioprotective signaling. This hormonal shift is known to trigger a cascade of metabolic alterations, including dyslipidemia, increased visceral adiposity, and autonomic dysfunction [9,10]. Consequently, physiological ovarian aging serves as a pacemaker for systemic senescence; in older women, this transition correlates with a loss of vascular elasticity and a heightened prevalence of heart failure and ischemic heart disease [11].

### 1.2. Preeclampsia as a Systemic Complication

PE is clinically defined by new-onset hypertension and end-organ damage after 20 weeks of gestation, rooted in defective placentation and subsequent systemic endothelial dysfunction. The pathogenesis is driven by a well-documented imbalance of pro-angiogenic and anti-angiogenic factors, such as soluble fms-like tyrosine kinase-1 (sFlt-1) [12,13]. While overt clinical symptoms typically subside postpartum, it is increasingly hypothesized that molecular “scars”—including persistent endothelial activation and subclinical vascular stiffness—remain. These systemic complications position PE not merely as an isolated obstetric event, but as a potential precursor to chronic vascular pathology that mirrors the premature cardiovascular aging seen in geriatric populations.

### 1.3. Accelerated Aging in Women with a History of Preeclampsia

While the epidemiological link between PE and future morbidity is clear, recent geroscience-informed research has begun to delineate the underlying mechanisms of accelerated biological aging. Epidemiological data confirm that women with a history of PE face a twofold increase in lifetime CVD risk and develop chronic hypertension nearly 7.7 years earlier than those with normotensive pregnancies [6]. Parallel to these clinical outcomes, emerging studies suggest a multisystemic decline: for instance, PE survivors demonstrate a reduced ovarian reserve, with AMH levels suggesting a reproductive age approximately 1.5 years older than age-matched peers [14].

Beyond the ovarian–vascular axis, preliminary evidence indicates that PE-induced systemic endothelial dysfunction may exert long-term effects on high-perfusion organs. PE is postulated to contribute to premature renal senescence, characterized by persistent podocyte loss and a faster decline in glomerular filtration rate (GFR) [15,16,17]. Furthermore, it is increasingly recognized as a potential catalyst for accelerated brain aging, associated with white matter hyperintensities and cognitive impairment [7,18,19]. The current mechanistic hypothesis suggests that this multi-systemic senescence is driven by common pathways, such as mitochondrial impairment, telomere shortening, and chronic inflammation [20,21,22], though further longitudinal studies are required to confirm these causal links.

However, this aging trajectory is highly dependent on the heterogeneity of PE phenotypes, where the timing of onset, severity, and underlying pathophysiology dictate the degree of biological insult. Furthermore, interpreting these outcomes requires caution due to potential confounders, such as baseline cardiovascular risk, genetic predisposition, and socioeconomic factors. These variables suggest that PE may not only act as a primary driver of senescence but also as a stress test that unmasks pre-existing vulnerabilities to accelerated aging.

## 2. Clinical and Epidemiological Evidence

### 2.1. Accelerated Ovarian Aging in Women with a History of Preeclampsia

Anti-Müllerian hormone (AMH) levels provide clinical evidence for the long-term decline in ovarian reserve following preeclampsia. AMH serves as a primary biomarker that indirectly reflects the size of the primordial follicle pool within the ovaries. Studies involving women with a history of PE have demonstrated significantly lower serum AMH levels compared to those who experienced normotensive pregnancies [8,14]. When this decline in AMH was quantified by mapping it onto age-specific AMH curves of a reference population, it was suggested that the reproductive age of women with a history of PE was advanced by approximately 1.5 years compared to normal controls. This figure provides epidemiological evidence that PE is associated with measurable and long-lasting damage to a woman’s ovarian biological clock, thereby accelerating the depletion of the ovarian reserve.

Interestingly, some longitudinal studies have reported that age-adjusted AMH levels measured prior to pregnancy do not serve as significant independent predictors of PE onset [23]. Specifically, the impact of pre-pregnancy AMH quartiles on the progression to PE was not significant after adjusting for age, BMI, and smoking history. The accelerated ovarian aging observed in women with a history of PE is attributed not to a pre-existing low baseline reserve, but rather to the direct insults of systemic vascular stress experienced during the pathological pregnancy. Since PE itself is regarded as a clear manifestation of impaired vascular health, this stress-induced damage occurring during or after pregnancy is hypothesized to support the “Ovarian–Vascular Axis” hypothesis [4,24].

While these findings point to a clear association, the interpretability of this accelerated decline must account for potential selection bias in clinical cohorts and the challenge of distinguishing direct causal insults from residual confounding factors that may independently influence both ovarian reserve and pregnancy outcomes.

### 2.2. Early Onset of Cardiovascular and Metabolic Diseases in Women with a History of Preeclampsia

Parallel to accelerated ovarian aging, it has been observed that systemic vascular aging and the risk of chronic diseases are also remarkably accelerated following PE [3,25] (Table 1). Epidemiological studies consistently suggest that a history of PE doubles a woman’s lifetime risk of cardiovascular disease (CVD). This CVD risk is especially pronounced in cases involving severe PE, early-onset PE developed prior to 34 weeks of gestation, or recurrent hypertensive disorders of pregnancy (HDP). According to large-scale cohort studies, women who experienced HDP were diagnosed with chronic hypertension an average of 7.7 years earlier than women without pregnancy complications [6]. This gap of over seven years offers indirect evidence that HDP potentially accelerates or unmasks a latent predisposition to systemic vascular aging, rather than acting as a discrete risk factor. Within the framework of geroscience, PE may be viewed as a physiological stressor that reflects the long-term trajectory of vascular health.

Furthermore, vascular damage persists long after delivery. PE survivors have exhibited persistently abnormal brachial artery flow-mediated dilation (FMD) for up to three years postpartum [26,27]. FMD is a primary measure of endothelial function, and its dysfunction is a potent predictor of CVD. Analysis has shown that women with endothelial dysfunction have nearly a tenfold increased risk of experiencing a subsequent fatal cardiovascular event compared to those without. This evidence of persistent vascular injury suggests that the residual effects of PE maintain an accelerated aging progression across both the ovaries and the systemic vasculature over the long term. These observations highlight the potential value of considering long-term CVD prevention and management strategies early in the clinical follow-up period.

Furthermore, the clinical significance of these long-term risks should be weighed against the heterogeneity of PE phenotypes, as the trajectory of vascular aging is likely modified by the severity of the initial insult and pre-existing maternal predispositions that act as shared drivers for both PE and chronic disease.

**Table 1:** Long-term Clinical Implications and Evidence of Accelerated Biological Aging Across Multiple Systems Following PE.

Target System	Clinical Implications & Evidence of Accelerated Aging
Cardiovascular	• Average of 7.7 years earlier onset of chronic hypertension in women with HDP history [6].
	• Elevated long-term risk of chronic hypertension and cardiovascular disease (CVD) [27].
	• Accelerated vascular aging driven by persistent vascular dysfunction [26].
Neurological (Brain)	• Evidence of accelerated brain atrophy and impaired brain health in midlife following HDP [18].
	• Persistence of structural and functional brain alterations years after delivery [7].
	• Significantly increased risk of future neurological disorders and cognitive impairment [19].
Reproductive (Ovary)	• Reduction in ovarian reserve and significantly lower AMH levels following preeclampsia [14].
	• Clinical evidence supporting accelerated ovarian senescence as a long-term sequel of HDP [8].
	• Increased risk of premature menopause and substantial shortening of the reproductive lifespan [23].
Renal (Kidney)	• Marked increase in the long-term risk of developing chronic kidney disease (CKD) [16].
	• Early manifestation of renal dysfunction consistent with cellular and organ-level aging [16].
	• Persistent injury to endothelial and glomerular structures post-preeclampsia [17].

## 3. Shared Pathogenic Mechanisms of PE and Ovarian Aging

**Figure 2:**
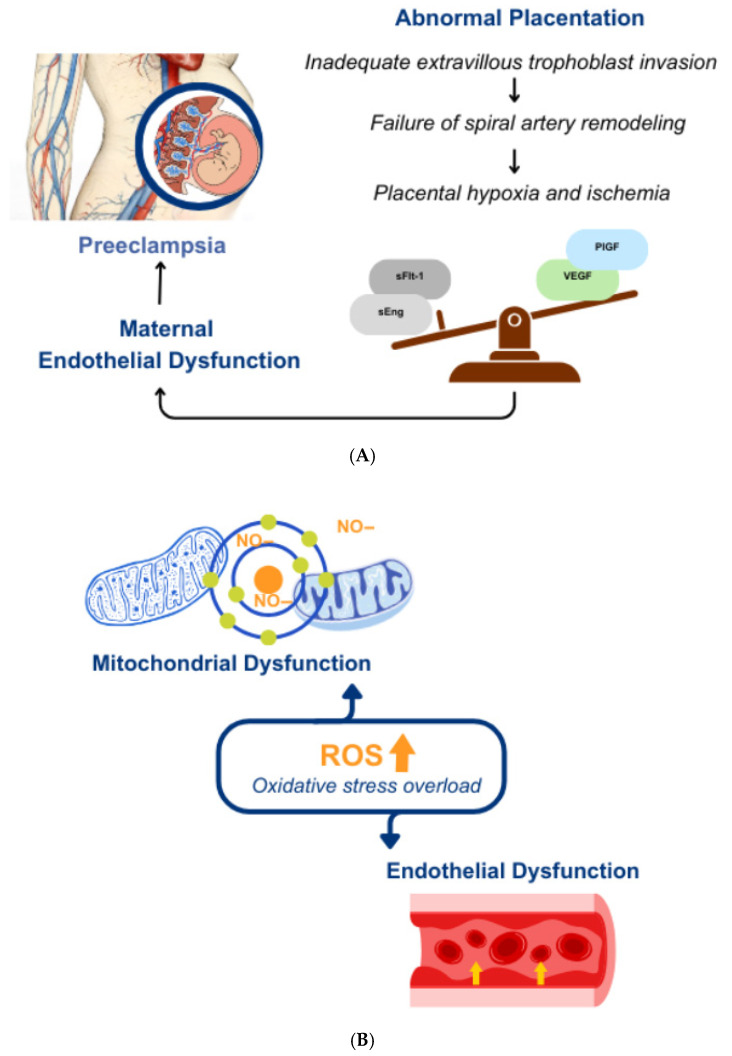

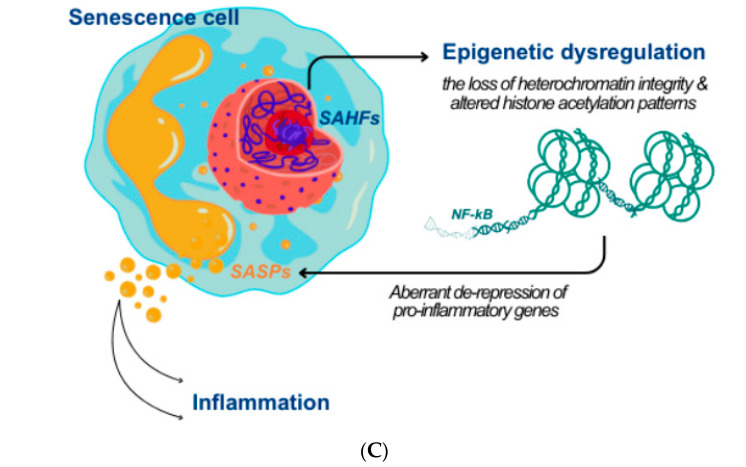
Shared molecular hallmarks of preeclampsia (PE) and ovarian aging. The schematic illustrates the mechanistic convergence between PE-related vascular stress and accelerated reproductive senescence. (**A**) The ovarian-vascular axis and endothelial dysfunction. Angiogenic Imbalance. Systemic elevation of sFlt-1 and sEng antagonizes VEGF/PlGF signaling, thereby inducing ovarian microvascular dysfunction and follicular ischemia. (**B**) Mitochondrial dysfunction and oxidative stress overload. Oxidative Stress and Mitochondrial Dysfunction. Excessive production of reactive oxygen species (ROS) and nitric oxide (NO) radicals triggers mitochondrial DNA damage and proteotoxic stress, ultimately impairing oocyte quality. (**C**) Cellular senescence, SASP, and epigenetic dysregulation. Cellular Senescence and Epigenetic Remodeling. Senescence-induced epigenetic dysregulation—characterized by the loss of heterochromatin integrity and SAHF formation—leads to the aberrant de-repression of pro-inflammatory genes. This activates the senescence-associated secretory phenotype (SASP), fostering a pro-inflammatory microenvironment that exacerbates ovarian aging.

The link between PE and accelerated ovarian aging is driven by a mechanistic crosstalk of shared molecular pathways, notably angiogenic imbalance, mitochondrial dysfunction, and impaired cellular stress responses [28,29] (Figure 2). Accumulating evidence suggests that this mechanistic convergence drives the accelerated biological aging observed in women with a history of PE, positioning the disorder as a pivotal early-life stressor within a geroscience framework.

### 3.1. The Ovarian–Vascular Axis and Endothelial Dysfunction

The fundamental pathogenesis of PE originates from compromised vascular health, primarily driven by the systemic release of placenta-derived anti-angiogenic factors. Specifically, soluble fms-like tyrosine kinase-1 (sFlt-1) acts as a potent antagonist that sequesters pro-angiogenic factors, such as vascular endothelial growth factor (VEGF) and placental growth factor (PlGF), thereby inducing widespread endothelial dysfunction [30,31]. Notably, the sFlt-1/PlGF ratio remains elevated in the PE group for up to one year postpartum and correlates positively with markers of arterial stiffness. This persistence indicates that the vascular injury associated with PE is not a self-limiting gestational event but may represent a prolonged pathological state extending well beyond delivery.

This systemic vascular insult may have direct and deleterious consequences for the ovaries. The ovaries are among the most highly vascularized organs in the female body, where the maintenance of the primordial follicle pool and subsequent follicular growth depend on an exquisitely regulated microvascular network. Given that PE represents a clinical manifestation of systemic vascular fragility, it has been hypothesized to function as a biological accelerator of premature ovarian aging [32,33,34]. Compromised vascular integrity disrupts the delicate ovarian microcirculation, exposing follicular cells to ischemia and nutrient deprivation. Such microenvironmental stress promotes follicular atresia and may contribute to the premature depletion of the ovarian reserve, potentially reflected clinically by reductions in anti-Müllerian hormone (AMH) levels and earlier onset of reproductive senescence.

Furthermore, circulating anti-angiogenic factors such as sFlt-1 may directly target ovarian endothelial cells and granulosa cells. Experimental evidence suggests that these factors can induce cellular senescence or apoptosis in critical follicular components, thereby disrupting molecular signaling pathways essential for oocyte survival. Consequently, a history of PE may initiate a cascade of follicle loss mediated by toxic circulating factors within the ovarian–vascular axis [35]. However, it should be noted that much of the mechanistic insight into these processes is derived from animal models and in vitro studies, underscoring the need for longitudinal human investigations to confirm causality and clinical magnitude.

### 3.2. Mitochondrial Dysfunction and Oxidative Stress (OS) Overload

A pivotal pathophysiological hallmark shared by PE and ovarian aging is mitochondrial dysfunction accompanied by oxidative stress (OS) overload [36,37]. In PE, defective trophoblast invasion results in hypoxia–reperfusion injury, triggering substantial oxidative damage within the placenta. This ischemic environment promotes excessive production of mitochondrial reactive oxygen species (mtROS) and the release of cell-free mitochondrial DNA (mtDNA) into the systemic circulation. These mtDNA fragments act as damage-associated molecular patterns (DAMPs), further amplifying endothelial dysfunction and reinforcing the PE phenotype.

Oxidative stress is likewise central to ovarian aging, which is characterized by a critical imbalance in which ROS levels overwhelm antioxidant defenses [38]. OS accelerates the aging of the oocyte pool by promoting apoptosis, telomere attrition, and oxidative damage to biological macromolecules. Oocytes are particularly vulnerable to these insults; excessive ROS can impair meiotic spindle formation and oocyte maturation, ultimately reducing reproductive longevity and diminishing ovarian reserve.

Within this context, PE can be conceptualized as an acute, high-intensity geriatric stressor that imposes a substantial oxidative burden on the female body during a critical reproductive window. The systemic mitochondrial dysfunction and OS overload observed during PE may damage the highly sensitive mitochondrial networks and DNA repair machinery of oocytes and granulosa cells, thereby physiologically hastening ovarian reserve depletion. These mechanisms provide a plausible biological link between pregnancy-related vascular stress and later-life reproductive aging.

### 3.3. Cellular Senescence, SASP, and Epigenetic Dysregulation

Ovarian aging involves a multifaceted decline characterized by cumulative DNA damage and the progressive accumulation of senescent cells. A central driver of this process is the failure of DNA damage repair (DDR) mechanisms [39,40]. The oxidative stress and mitochondrial injury induced during PE may exacerbate DNA damage, leading to activation of senescence-associated markers such as p16INK4a and p21, which enforce irreversible cell-cycle arrest.

Crucially, this process is accompanied by significant epigenetic dysregulation. In senescent cells, the loss of heterochromatin integrity and altered histone acetylation patterns—such as the formation of senescence-associated heterochromatic foci (SAHF)—lead to the aberrant de-repression of pro-inflammatory genes [41]. This epigenetic remodeling serves as a molecular switch that triggers and sustains the senescence-associated secretory phenotype (SASP), characterized by the secretion of pro-inflammatory cytokines (e.g., IL-6, TNF-alpha), chemokines, and matrix metalloproteinases [42].

In PE survivors, the persistence of these SASP-related mediators does not remain localized; rather, they are released into the systemic circulation, fostering inflammaging—a state of chronic, low-grade systemic inflammation [43]. This transition from cellular secretion to a systemic environment occurs as SASP factors recruit immune cells and induce secondary senescence in neighboring healthy tissues, a phenomenon known as the “bystander effect.”

This inflammatory milieu not only contributes to progressive vascular stiffening but may also degrade the ovarian microenvironment, establishing a feed-forward loop that accelerates both systemic aging and reproductive senescence. These shared inflammatory and senescent pathways may ultimately converge on the heightened cardiovascular vulnerability observed in aging women with a history of PE, reinforcing the relevance of PE as an early-life determinant of late-life cardiovascular disease.

Emerging evidence further points to overlapping biomolecular signatures in the form of microRNAs (miRNAs). Specific circulating miRNAs, including inflammation-associated miRNAs (inflamma-miRs) that regulate immune signaling and cell death pathways, are dysregulated in both PE and premature ovarian insufficiency (POI) [44,45,46]. Identification of these shared miRNA profiles in PE survivors may offer insight into their future risk of POI and accelerated aging, with potential utility as non-invasive diagnostic biomarkers or targets for geroprotective interventions.

## 4. Geroprotective Intervention Strategies to Modify Aging Pathways in Women

### 4.1. A Paradigm Shift: PE as a Catalyst for Accelerated Biological Aging

The multi-axial acceleration of biological aging observed in women with a history of preeclampsia (PE) calls for a paradigm shift in long-term clinical management. PE should be reconsidered not merely as a transient pregnancy complication, but as a pivotal early-life biological stress event that may influence a woman’s overall healthspan. Under the geroscience approach, aging is defined as the shared biological substrate underlying multiple chronic diseases, and interventions targeting fundamental aging pathways are proposed to yield simultaneous benefits across organ systems. From this perspective, the multisystemic decline spanning ovarian, cardiovascular, metabolic, and neurocognitive functions observed after PE is unlikely to represent isolated organ injuries, but rather the consequence of an accelerated and asymmetric activation of aging-related biological programs.

### 4.2. Precision Risk Stratification via Aging Biomarkers

As this multisystemic decline reflects a fundamental aging process, defining the aging profile through a precision risk-stratification model is essential for individualized care. An effective model should integrate biomarkers of fundamental aging biology with conventional clinical indicators to provide a more comprehensive risk profile. Epigenetic clocks [47,48,49], including the Horvath clock and GrimAge, enable the direct quantification of biological age acceleration through DNA methylation patterns, while circulating pro-inflammatory cytokines (IL-6, CRP, TNF-α) and senescence-associated secretory phenotype (SASP)-related factors reflect the systemic onset of inflammaging. In parallel, mitochondrial function indicators (mtDNA copy number, GDF15, and FGF21), along with cellular senescence markers like p16INK4a and p21, serve as robust parameters for assessment. These markers reflect functional impairment along the ovarian–vascular axis, which acts as the central pathophysiological interface linking reproductive aging with cardiovascular vulnerability. Collectively, these biomarkers may support a novel classification framework that stratifies post-PE women into normal, borderline, and high-risk aging phenotypes, thereby enhancing the clinical feasibility of personalized geroprotective interventions.

In the long-term care of women with a history of PE, precise monitoring of ovarian aging is pivotal, as it serves as a primary driver that accelerates systemic aging through hypoestrogenism following menopause. Conventional ovarian reserve assessments based on anti-Müllerian hormone (AMH) levels and antral follicle count (AFC) often overlook the underlying structural and functional decay in ovarian tissue, including reduced microvascular density, stromal fibrosis, and mitochondrial dysfunction [50,51]. Accordingly, the conceptual development of an Ovarian Aging Biomarker (OAB) Index—which integrates conventional measures like AMH and AFC with imaging-based morphological indicators, vascular signatures, and fibrosis-related parameters—may offer a more comprehensive assessment of ovarian biological senescence. While such a composite index remains hypothetical and requires rigorous validation, it illustrates a framework through which reproductive aging can be quantitatively aligned with systemic vascular and metabolic aging. Given the persistent elevation in cardiometabolic risk for decades following PE, longitudinal monitoring of cardiovascular and metabolic health is essential throughout the life course.

### 4.3. Nonpharmacological Lifestyle Interventions

Non-pharmacological interventions currently represent the most evidence-supported strategies within geroscience-informed care. Regular aerobic and resistance exercise has been consistently associated with mechanisms that decelerate biological aging, including enhanced mitochondrial biogenesis, attenuation of systemic inflammation, and improved insulin sensitivity [52,53]. Similarly, anti-inflammatory and metabolically stabilizing dietary patterns contribute to the preservation of endothelial function and vascular elasticity [54,55,56]. Recognizing that the management of accelerated aging after PE is a protracted lifelong journey, patient-led lifestyle interventions become a pivotal axis of care. These strategies are not only immediately translatable but also offer a multifaceted protective shield against various age-related disorders, empowering women to actively mitigate their cumulative aging burden.

### 4.4. Pharmacological Gerotherapeutics

Pharmacological geroprotective strategies span a wide range of translational stages, from agents with established cardiovascular indications to experimental compounds targeting core aging mechanisms. Metformin represents a leading candidate for drug repurposing, given its capacity to activate AMP-activated protein kinase (AMPK), alleviate mitochondrial stress, and potentially delay epigenetic aging [57,58]. Statins, widely used for cardiovascular risk reduction, exert pleiotropic anti-inflammatory and endothelial-stabilizing effects and have been explored in both the prevention and treatment of PE [59,60,61]. In addition, SGLT2 inhibitors may represent novel candidates through their combined effects on metabolic homeostasis and renovascular protection [62,63]. However, while clinical trials utilizing these repurposed drugs are currently underway for a broad range of age-related indications, no pharmacological agent has yet received regulatory approval specifically for modulating biological aging.

Emerging classes of gerotherapeutics, including senolytics (e.g., dasatinib and quercetin) and senomorphics (e.g., rapalogs), have demonstrated proof-of-concept efficacy in preclinical models by modulating senescent cell burden and restoring cardiovascular and metabolic function [64,65,66]. Nevertheless, most of these compounds remain in the preclinical stage based on animal models, and their translation to human application will require extensive research and time. During this translational process, a rigorous evaluation of safety and clinical utility is paramount—not only for older populations but especially for women of reproductive age.

While these agents provide a conceptual framework for understanding PE as a model of accelerated biological aging, none of these pharmacological approaches is currently indicated specifically for post-PE women. Therefore, meticulous validation of safety, optimal timing, and efficacy—particularly concerning future fertility and long-term reproductive health—through prospective randomized clinical trials is essential before these therapies can be considered for clinical implementation.

In conclusion, geroscience-based intervention strategies offer a fundamental expansion of traditional obstetric paradigms, providing a unifying framework for addressing both reproductive and systemic aging acceleration following PE. The integration of aging biomarker-driven risk stratification, early ovarian-vascular interventions, and the judicious use of gerotherapeutics serves as the primary engine for enhancing long-term healthspan in women following PE. Future research should include (1) refinement of predictive models using high-resolution aging biomarkers, (2) development of dynamic biological feedback markers to assess intervention efficacy in real time, and (3) establishment of dedicated clinical trial platforms to validate the safety and translational potential of geroprotective therapies. Collectively, these efforts have the potential to align post-PE clinical care with a geroscience-centered approach, ultimately fostering more proactive prevention of late-life cardiovascular disease.

## 5. Conclusions

Emerging evidence mandates a redefinition of preeclampsia (PE) beyond a transient pregnancy complication. PE serves as a potent early-life stress test that triggers an accelerated aging path within integrated reproductive and cardiovascular systems. This review highlights that women with a history of PE experience measurable acceleration of ovarian aging, reflected by reduced ovarian reserve and earlier reproductive senescence, alongside a substantially increased risk of premature cardiovascular and metabolic disease that manifests later in life, particularly during the postmenopausal and geriatric transitions.

This shared progression is anchored by the ovarian–vascular aging axis. Within this framework, molecular drivers including endothelial dysfunction, mitochondrial stress, and cellular senescence converge to trigger concurrent functional decay across these systems. These mechanisms align closely with the established hallmarks of biological aging, positioning PE within a geroscience paradigm as a female reproductive model of accelerated aging.

From a clinical standpoint, this paradigm shift carries significant implications for life-course health management. Incorporating reproductive history—particularly a history of PE—into long-term cardiovascular risk assessment offers a unique opportunity for the earlier identification of women on an accelerated aging trajectory. Biomarker-based stratification using epigenetic clocks, inflammatory and senescence-associated markers, and mitochondrial indicators may enable precision surveillance well before overt cardiovascular disease develops. Such an approach aligns with the goals of preventive cardiology in aging populations and bridges the long-standing gap between obstetric surveillance and geriatric preventive medicine, fostering a necessary continuum of care.

Moreover, this review underscores the potential of geroprotective strategies to decelerate the accelerated aging trajectory following PE. The synergy of lifestyle modifications, intensive cardiometabolic risk management, and targeted geroprotective agents constitutes the strategic foundation for mitigating the cumulative aging burden in post-PE women. While emerging gerotherapeutics remain largely experimental and require rigorous validation through prospective human trials, they provide a compelling translational framework for future research aimed at reducing late-life cardiovascular morbidity in women exposed to early-life vascular stress.

In summary, redefining preeclampsia as an early-life driver of reproductive and cardiovascular aging reframes it as a pivotal opportunity for lifelong prevention, shifting the focus from a past obstetric event to future systemic health.

## Data Availability

No new data were created or analyzed in this study. Data sharing is not applicable to this article.
